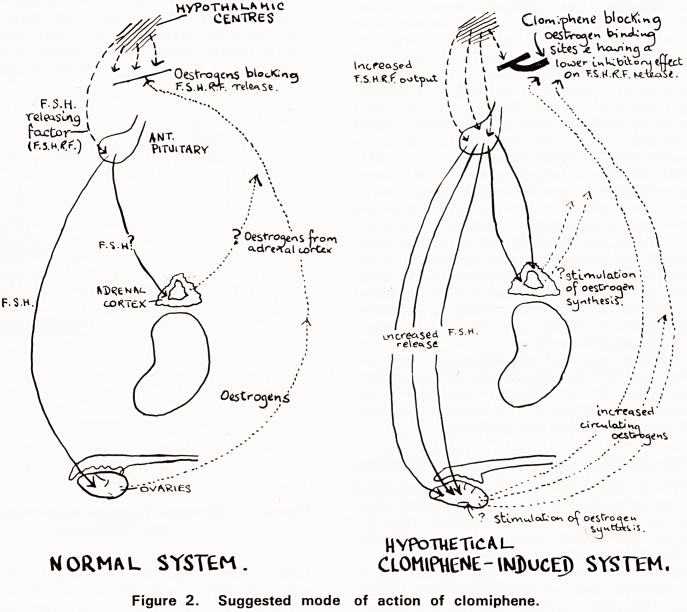# The Development and Usage of Clomiphene

**Published:** 1972-10

**Authors:** Ingrid M. Bowdler

**Affiliations:** Clomiphene is an oestrogen-like compound discovered thirteen years ago and is now widely used for promoting ovulation in the treatment of infertility


*Bristol Medico-Chirurgical Journal Vol. 87*

### The Development and Usage of Clomiphene by

**Ingrid M. Bowdler,** Medical Student

Clomiphene is an oestrogen-like compound discovered thirteen years ago and is now widely used for promoting ovulation in the treatment of infertility.

### DEVELOPMENT

In the early 1960's methods of promoting conception were based on therapy with compounds similar to follicle stimulating hormone (F.S.H.) and luteinizing hormone (L.H.) All were very expensive, had to be given by injection, and could lead to multiple pregnancies as individual sensitivity to them varied greatly. Interest in clomiphene was instigated when a similar compound MER-25, was given to four patients with Stein-Leventhal syndrome and effected a return to normal hormonal changes and ovulation (Kistner and Smith, 1959). Clomiphene itself was first investigated by Holtkamp et al (1960) who found that it stopped the oestrous cycle of mature female rats and hence acted as a contraceptive in these animals. It was hoped that this compound would act similarly in the human female. A year later, however, Greenblatt et al (1961) reported that clomiphene induced ovulation in women. This study was on four women with a normal ovulatory pattern, three girls with precocious puberty and forty-three women with primary or secondary amenorrhoea, and revealed that the drug had no marked effect on the women and young girls with established menstrual cycles nor on those with primary amenorrhoea. In those with secondary amenorrhoea or Stein-Leventhal syndrome and similar conditions however, ovulation was brought about in 77 per cent of cases of whom 12.1 per cent became pregnant.

The contrasting action of clomiphene on rats and humans was, and still is, unexplained. The possibility of a dosage controlled phenomenon (increased or decreased dosage causing the drug to have anti-fecundity properties) was investigated and disproved.

### STRUCTURE, METABOLISM AND MODE OF ACTION

Clomiphene is structurally similar to the long-acting oestrogen chlorotrianisene ("Tace") and slightly similar to the synthetic oestrogen stilboestrol (Fig 1). If stilboestrol can occupy oestrogen receptor sites, although structurally unlike the steroid molecule, it seems reasonable to suppose that clomiphene can do so too. Clomiphene may act by binding with oestrogen receptor sites and, having a low intrinsic activity these slight oestrogenic effects may be brought about, while the anti-oestrogenic effects could be due to clomiphene blocking the natural hormone's access to its binding sites. It may indeed function by "tricking" the negative feedback system controlling oestrogen production into a response normally occurring when oestrogen levels are considerably lower. The exact site of action is unknown and, as each cell type with which oestrogen binds may have a different type of binding site, clomiphene may only "fit", and act, at a few of these sites.

Metabolism has been studied using radio-actively labelled carbon in the compound. Its half-life in the body is about five days and it is excreted unchanged, mainly in the faeces and to a lesser extent in urine. Elimination is delayed by enterohepatic circulation.

The site and mode of action of clomiphene is still speculative despite ten years of investigation. When released for clinical trial in 1961 it was noted to have a thermiogenic effect in the luteal phase of the cycle and hence the postulation was made that it might act via the hypothalamus, causing release of L.H. By 1966, however, there were two schools of thought—some

**Figure 1. Structural formulae of oestrone, stilboestrol and clomiphene.**

investigators thought that the drug acted on the pituitary, stimulating both the production of F.S.H. and L.H. (Crooke et al 1969), while others believed that it acted solely on the gonads causing an increased oestrogen synthesis (Smith 1966). To avoid cyclical hormonal changes this last postulate was studied in a rather unusual way. Clomiphene was given to normal men and the rise in urinary oestrogens noted during and after treatment, but with no alteration in gonadotrophin level, was taken to indicate that its action was solely on the gonads.

As the menstrual cycle is partly controlled by higher centres via the hypothalamus, the question arose whether ovulation occurred solely because of clomiphene's placebo effect. A number of double blind trials proved that this was not so (Johnson et al 1966).

In 1968 Keller et al performed separate bioassays of the F.S.H. and L.H. secreted. It was found that although total gonadotrophins did not alter appreciably, the L.H. level rose ten to twelve days after the beginning of a course of treatment, ovulation occurring a day or two after this rise. As clomiphene caused no alteration in urinary levels of F.S.H. and L.H. when given to women with ovarian dysgenesis however it was concluded that some ovarian function, either the formation of oestrogen precursors or oestrogen itself, was necessary before the drug could act.

Two years ago, two Yugoslavian workers (Kicovic and Subotic 1970) threw doubt on the postulate that the necessary oestrogens had to be synthetized in the ovaries by showing that there was a rise in oestrogen synthesis following clomiphene therapy in oophorectomized women. They suggested that the adrenal cortex might therefore be involved in the body's response to this drug.

*In vitro* studies show that clomiphene increases concentrations of triphosphopridine nucleotide, which has a catalytic effect on the aromatization reaction changing an androgen-type compound to an oestrogen (Hagerman et al 1966). It is not known, however, whether this is what occurs *in vivo* and in spite of several investigations this problem is still not satisfactorily solved.

### TREATMENT OF FEMALE INFERTILITY

**Dosage.** In the early trials dosage varied greatly as the best regime had not then been established. The usual dosage scheme now is 50 milligrammes orally for 5 days, and if no response to this is detected then a hundred milligrammes is given for 5 days in the next "cycle" of treatment. Higher doses than this are not given as they would increase the likelihood of side effects. Treatment begins on the first day of a period, if and when there is one (some people begin on the fifth day but this lengthens the cycle abnormally) or at any time if the woman is not menstruating. If no ovulation has occurred after two "cycles" of five day treatment with a hundred milligrammes per day it is unlikely to occur subsequently. Similarly if pregnancy does not occur after about six ovulatory cycles, it is unlikely to occur at all and repeat courses are seldom used for this reason.

Ovulation usually takes place between six and eleven days after such a five day course and can be recognized by alteration in the patient's basal body temperature taken daily. To avoid giving clomiphene in early pregnancy thirty days are usually left between

**Figure 2. Suggested mode of action of clomiphene.**

one treatment course and the next, unless the woman menstruates.

**Success.** For the patient, successful treatment is that which leads to pregnancy, and in those selected for this therapy the pregnancy rate is 20-25 per cent, different trials reporting varying figures. On a more scientific basis, however, the occurrence of ovulation has been equated with success the rate of which varies in different trials from 47 per cent to 81 per cent, depending mainly on the methods of patient selection.

The difficulties met with are exemplified in a study by Lamb and Guderian (1966), who used the following techniques:

(1) Monitoring of basal body temperature
(2) Cervical mucus examination every two to four days for:
   (a) quantity
   (b) ferning—occurs in oestrogen-primed cervical secretion, decreases with clomiphene treatment and increases at ovulation, as does
   (c) "Spinnbarkeit"—elasticity of cervical mucus
(3) Vaginal cytology, judging the karyopyknotic index.
(4) Ovarian size by bi-manual palpation.

Of these (1) and (2) emerged as the most accurate techniques, the direct effect of the drug altering (3) and (4) considerably.

Despite the high rate of ovulation caused, pregnancy rates are low. A number of hypotheses have been put forward to account for this (Whitelaw et al 1970).

(1) an increased abortion rate — possibly these women have a greater likelihood of hormonal inbalance in the early months of pregnancy;
(2) an altered rate of tubal transport—because of neuro-endocrine disturbance the blastocyst may not reach the uterine cavity at the optimum time for implantation;
(3) a change in cervical mucus—making sperm penetration more difficult.

In an attempt to decrease these factors progestogens are frequently given in the early part of the pregnancies which occur.

**Side Effects.** The side effects increase with the dosage given. In one study (Pildes 1965) approximately 40 per cent complained of lower abdominal pain and about the same percentage developed ovarian cysts. Ovarian enlargement and luteal cyst formation was found to be more frequent in cases of Stein-Leventhal syndrome. A few women also had hot flushes and visual disturbances, such as spots before the eyes or blurring of vision. No undesirable effects have been noted when the drug has inadvertently been given in early pregnancy.

The likelihood of multiple births occurring increases sixfold with clomiphene therapy, but usually only two ova are fertilized, the higher numbers of multiple births occurring mainly after human pituitary gonadotrophin treatment. Even careful dosage monitoring will not eradicate this problem as a tendency to multiple pregnancy is thought to be inherent in women with Stein-Leventhal syndrome.

**Patient Selection.** Before treatment is commenced a full clinical examination is made to exclude genetical or anatomical defects which may underlie the woman's infertility. Also, the husband is proven to be fertile. To exclude gynaecological disease a diagnostic curettage should also be done on these patients. Those women which respond best to treatment are those with Stein-Leventhal syndrome, metropathia haemorrhagica, and amenorrhoea because of marginal pituitary imbalance or insufficiency.

The presence of liver disease is a contraindication to the use of the drug because it is concentrated in the liver and excreted in bile. Otherwise the only contra-indication to use is excessive ovarian cyst formation. This is especially likely to happen in cases of Stein-Leventhal syndrome and usually regresses spontaneously once treatment is stopped, but may require androgen therapy.

Severe menorrhagia has also been treated with clomiphene, but its effect is frequently short-lived when the drug is discontinued and long-term therapy is not practised as little is known about the side-effects of chronic administration. The screening necessary before treatment and the side-effects, have not precluded the widespread use of clomiphene and, in spite of a sizeable failure rate, it has proved to be a very useful drug.

### USE IN MALE INFERTILITY

More recently interest has been directed towards the use of clomiphene in treating men with azoo- or oligospermia, in the hopes that increased interstitial cell stimulating hormone would be secreted, and would ameliorate the condition. In 1970, Palti published a study of infertile men who were treated with clomiphene. These men went through routine clinical and laboratory tests to exclude underlying pathology, and were given varying doses of clomiphene for periods varying from twenty to sixty days. The results showed that 47 per cent achieved slightly higher sperm counts and about the same percentage showed an increased sperm motility. Variations in the results between this and other trials are considerable however and, furthermore, no correlation between results and the dosage and duration of treatment has been found. No doubt more work will be done on this aspect in the future.

### COMBINED THERAPY WITH GONADOTROPHINS

Gonadotrophins alone are able to stimulate ovulation in women with very low or absent hormone levels on whom clomiphene has no effect. In 1969 Crooke et al showed that the incidence of ovulation was greater with combined therapy than with either treatment alone. Their result of a 173 per cent increased incidence of ovulation compared to that of gonadotrophin treatment alone is impressive and, although the patients in their trial were probably carefully selected, this technique may nevertheless be of value, especially in those cases where either therapy alone has failed.

### REFERENCES

Crook, A. C., Hanisotia, M. D., Bertrand, P. V. (1969) Lancet 1:587.

Greenblatt, R. B., Barfield, W. E., Junck, E. C., Roy, A. W. (1961) Journal of the American Medical Association 178: 101.

Hagerman, D. P., Smith, O. W., Day, C. F. (1966) Acta Endocrinologica, 51, 591.

Holt Kamp, D. E., Greslin, J. G., Root, C. A., Lerner, L. J. (1960) Proceedings of the Society of Experimental Biology, 105, 197.

Johnson, Cohen, Goldfarb, Rakoff, Kistner, Plotz, Vorys, (1966) International Journal of Fertility, 11. 265.

Keller, P. J., Neville, A. H., Wyss, H. I. (1968) Fertility and Sterility, 19, 892.

Kicovic, P., Subotic, Z. (1970) American Journal of Obstetrics and Gynecology, 108: 1077.

Kistner, R. W., Smith, O. W. (1959) Surgical Forum 10: 725.

Lamb, E. J., Guderian, A. M. (1966) Obstetrics and Gynecology, 28, 505.

Palti, Z. (1970) Fertility and Sterility, 21, 838.

Pildes, R. B. (1965) American Journal of Obstetrics and Gynecology, 91, 466.

Smith, O. W. (1966) American Journal of Obstetrics and Gynecology, 94, 440.

Whitelaw, M. J., Kolman, C. F., Grams, L. R. (1970) American Journal of Obstetrics and Gynecology, 107, 865.

## Figures and Tables

**Figure 1. f1:**
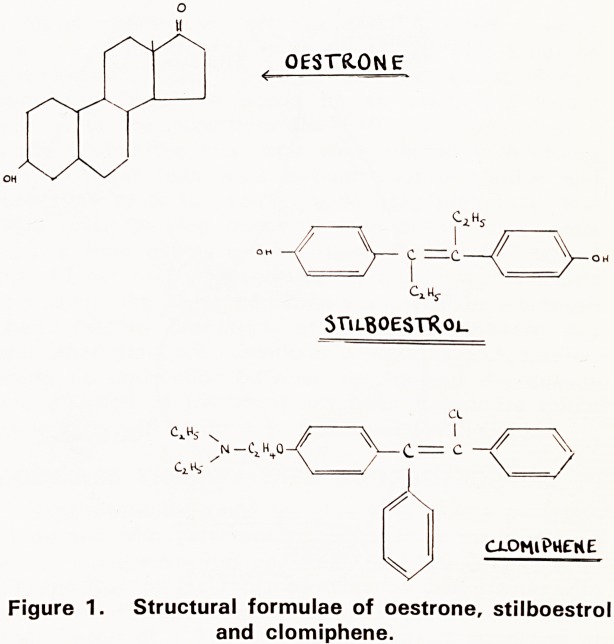


**Figure 2. f2:**